# Analgesic and Anti-Inflammatory Properties of Ethanolic Extract of *Piper vicosanum* Leaves

**DOI:** 10.3390/pharmaceutics14112455

**Published:** 2022-11-14

**Authors:** Armando Jorge Junior, Joyce dos Santos Lencina, Elisangela dos Santos, Jonas da Silva Mota, Roberto Kenji Nakamura Cuman, Elisabete Castelon Konkiewitz, Cândida Aparecida Leite Kassuya, Saulo Euclides Silva-Filho

**Affiliations:** 1 Health Sciences College, Federal University of Grande Dourados, Dourados 79804-970, MS, Brazil; 2 Pharmaceutical Sciences, Food and Nutrition College, Federal University of Mato Grosso do Sul, Campo Grande 79070-900, MS, Brazil; 3 Natural Resources Study Center, State University of Mato Grosso do Sul, Campo Grande 79804-970, MS, Brazil; 4 Department of Pharmacology and Therapeutics, State University of Maringá, Maringá 87020-900, PR, Brazil

**Keywords:** *Piper vicosanum*, inflammatory response, articular inflammation, nociception

## Abstract

Nonclinical trials are important to validate the efficacy and safety of medicinal plants. Scientific toxicological studies with *Piper vicosanum* Yuncker have showed its safety; however, no studies have indicated the analgesic or antiarthritic potential of the ethanolic extract of *P. vicosanum* leaves (EEPV). The objective of the present work was to evaluate the antiarthritic and antinociceptive effects of EEPV in experimental mouse models. The oral administration of EEPV (100, 300, and 700 mg/kg) and dexamethasone (1 mg/kg) were performed in carrageenan-induced pleurisy, in formalin and acetic-acid-induced nociception, and in zymosan-induced articular inflammation models in Swiss mice. The EEPV (300 mg/kg) was tested in zymosan-articular inflammation, the complete Freund’s adjuvant (CFA) inflammatory model, and in in situ intravitreal microscopy analysis of rolling and adhesion events of leukocytes in the mesenteric microcirculation in mice. EEPV significantly inhibited: (i) nociceptive response at phase 1 and 2, and also in the cold response in the formalin model; (ii) abdominal contortion induced by acetic acid; (iii) mechanical hyperalgesia after 4 and 6 h, knee edema after 6 h, and leukocyte migration in articular inflammation induced by zymosan. All doses of EEPV reduced the leukocyte migration to the inflamed pleural cavity and knee edema 4 h after the zymosan knee injection. The treatment with the EEPV significantly inhibited the CFA-induced edema, mechanical and cold hyperalgesia, and NAG and MPO. The EEPV also significantly inhibited carrageenan-induced leukocyte rolling and adhesion. The present study revealed, for the first time, the antiarthritic and antinociceptive effects of the EEPV.

## 1. Introduction

The Brazilian National Health Surveillance Agency ensures the population’s health by guaranteeing the safety of products and services, including the medicinal plants and phytotherapeutic products [1,2]. The investigation of pharmacological activities of natural products, obtained from medicinal plants, is essential in the search for new alternatives for the treatment of diseases, reducing consumption, and, consequently, the incidence of adverse effects of traditionally used drugs. The nonclinical and clinical trials can show the safety and efficacy of new herbal products through toxicological studies that investigate the safety in the use of these products [3].

*Piper vicosanum* Yuncker, a plant of Piperaceae family, is a little-explored species that has shown a great therapeutic potential. Several plants of *Piper* genus are used as food and in folk medicine to relieve bronchitis, intestinal pain, inflammatory conditions, and pain [4]. The composition of the essential oil obtained from *P. vicosanum* Yuncker leaves collected in the Brazilian Atlantic Forest [5] and in Cerrado [6] were investigated. Both oils obtained in these studies were rich in monoterpenoids, such as limonene and other compounds [5,6].

In a toxicological analysis of the ethanolic extract of *P. vicosanum* leaves (EEPV), Wistar rats during a 28-day treatment with EEPV did not exhibit clinical signs of toxicity. However, some biomarkers of renal and liver functions were altered [4]. The antiedematogenic effects of essential oil from *P. vicosanum* leaves (100 and 300 mg/kg) were revealed, and the anxiolytic effect of EEPV was demonstrated, and this essential oil did not present in vivo toxicity [6,7].

Since only few scientific studies related to the anti-inflammatory activity of the extract of *P. vicosanum* were performed, the objective of this work was to evaluate its analgesic, anti-inflammatory, and antiarthritic potential. The ethanolic extract from *Piper vicosanum* leaves (EEPV) was tested in several models of experimental inflammation and nociception, such as carrageenan-induced pleurisy, in formalin and acetic-acid-induced nociception, and in situ mesenteric bed microcirculation models. The antiarthritic and anti-inflammatory potential of EEPV was assayed in zymosan-induced articular inflammation and CFA-induced persistent inflammation models in mice.

## 2. Materials and Methods

### 2.1. Preparation of the Ethanolic Extract of P. vicosanum Leaves (EEPV)

The species *P. vicosanum* was collected in February 2017 in Dourados, MS, Brazil (coordinates 22°12′37.8′′ S, 54°55′2.6′′ W), and the specimen was deposited in the Federal University of Grande Dourados (UFGD) herbarium (DDMS 4411). In addition, the use of the species was registered and approved by the National System for the Management of Genetic Heritage and Associated Traditional Knowledge (SisGen Registry number AE032EB).

Leaves (1430 g) were dried at room temperature, pulverized, and subjected to three extractions by maceration with 70% ethanol for 7 days. The extract was concentrated in a reduced-pressure rotary evaporator and dried in a hood [7]. A total of 203 g of ethanolic extract were obtained, as previously described.

The phytochemical analysis was previously described in a study carried out by our group [7], in which the presence of alkaloids, phenolic compounds, tannins, steroids, and triterpenes was identified. In the present work we carried out the determination of the content of phenolic compounds and flavonoids, and we performed the phytochemical analysis of EEPV by HPLC-DAD.

### 2.2. Determination of the Content of Phenolic Compounds

For each 100 μL of sample, 1.5 mL of 2% sodium carbonate aqueous solution, 0.5 mL of Follin–Ciocalteau reagent (1:10 *v/v*), and 1 mL of distilled water were added. It was allowed to react for 30 min, and the reading was performed in the UV-VIS Global Trade Technology spectrophotometer at a wavelength of 760 nm. The same procedure was performed for the blank, replacing 100 μL of the sample with 100 μL of the solvent used in the preparation of the solutions. The concentration was calculated by preparing an analytical curve, using gallic acid as a standard. With the data, the linear regression was developed and the straight-line equation was obtained, which had the data used in the calculation of the real samples. The result was expressed in mg of gallic acid per g of lyophilized extract. From these readings taken in the spectrophotometer, an average was taken, and the data were obtained through the formula: y = a + bx, in which “y” refers to the reading made in the spectrophotometer, “x” refers to the phenolic, “a” refers to white (0.0118), and “b” to a constant, with “a” and “b” being related to gallic acid.

### 2.3. Determination of Flavonoid Content

For each 500 μL of sample, 1.5 mL of 95% ethyl alcohol, 100 μL of 10% aluminum chloride, 100 μL of 1 mol·L^−1^ sodium acetate, and 2.8 mL of distilled water were added. It was allowed to react at room temperature for 40 min and the reading was performed on a UV-VIS Global Trade Technology spectrophotometer at a wavelength of 415 nm. The same procedure was performed for the blank, replacing 500 μL of the sample with 500 μL of the solvent used to prepare the solutions. The concentration was calculated by preparing an analytical curve, using quercetin as a standard. With the data, the linear regression was developed and the equation of the line was obtained, which had the data used in the calculation of the real samples. The result was expressed as mg of quercetin per g of lyophilized extract. By means of the readings taken in the spectrophotometer, an average was performed, and these data were calculated according to the formula: y = a + bx, where “y” refers to the reading made in the spectrophotometer, “x” refers to the phenolic content, “a” refers to blank (0.0121), and “b” to a constant.

### 2.4. Phytochemical Analysis by HPLC-DAD

The analysis was performed on a VARIAN chromatograph with a ternary pump system, model 210, diode array detector (DAD), with scanning between 200 and 800 nm, programmed at 254 nm. A Phenomenex C-18 column (4.6 mm × 250 mm, particle diameter 10 µm) and precolumn (25 mm × 3 mm) of the same phase as the column was used. Elution was performed in a gradient system: MeOH/H_2_O from 5 to 100% methanol, taking 20 min to reach 100% methanol, 5 min to 100% methanol, and 5 min to return to the initial condition. The analysis time was 25 min. Pump flow rate of 1 mL/min and injected volume of 10 µL. Samples were filtered with a 0.20 µm microfilter.

### 2.5. Animals

Male and female *Swiss* mice (weight between 25 and 30 g) were obtained from UFGD central biotherium. These animals were transferred to the sectorial biotherium of the Faculty of Health Sciences (FCS) of UFGD. They were kept in propylene boxes and maintained in controlled light conditions (12 h light/dark) and temperature (mean 23 ± 2 °C), receiving water and commercial feed ad libitum. On the day of the experiment, they were taken to the laboratory for adaptation 60 min before the start of the experiment. Anesthesia was induced with 20 μL of 10 mg/kg xylazine 2% + 100 mg/kg ketamine 10% intraperitoneally. When the animals were to be euthanized, we used a solution of 100 µL xylazine 2% + 100 µL of ketamine 10% intraperitoneally. The experiment was conducted according to the animal rights guidelines with the approval of the UFGD Committee on Animal Ethics under number 37/2017.

### 2.6. Formalin-Induced Nociception Test

For the formalin test, mice were divided in 5 groups of 6 mice each. The experimental groups were treated with oral EEPV (100, 300 and 700 mg/kg); the vehicle group was treated with oral sterile saline (0.9%) and the morphine group was treated with 5 mg/kg subcutaneously. After 60 min of administration of the treatment, the animals received intraplantar injections of formalin 2.5% (20 μL/animal) in the right rear paw. After administration of formalin, the animals were placed individually under a glass funnel, and the time the animals spent licking the right hind paw was timed in two phases (phase I: 0 to 5 min; phase II: 15 to 30 min). Time zero was considered immediately after formalin injection. At the end of phase 2 (30 min), cold sensitivity tests (allodynia) were performed with the application of 20 μL of acetone topically on the right hind paw. After 60 min, paw edema analysis was performed using a plethysmometer (Insight^®^). At the end, the animals were euthanized [8].

### 2.7. Acetic Acid-Induced Abdominal Writhing Test

For the abdominal writhing test, the animals were distributed in 5 groups of 6 mice each. The EEPV groups were treated with oral doses of 100, 300, and 700 mg/kg. The vehicle group was treated with sterile saline solution (0.9%) and the morphine group was treated with 5 mg/kg subcutaneously. After 60 min, we administered acetic acid 0.8% (0.1 mL/10 g) intraperitoneally and observed behavior for over 20 min [9].

### 2.8. Carrageenan-Induced Pleurisy

The male mice were distributed in six groups of six mice each. The control and naïve groups were treated with saline solution (0.9%), the groups were treated with oral EEPV at 100, 300, and 700 mg/kg, and the reference group received dexamethasone at 1 mg/kg by the subcutaneous route (s.c.). All the animals (except the naïve group, which received 100 µL of sterile saline 0.9% intrapleural) received 100 µL intrapleural injection of carrageenan 1% (500 µg/cavity) 60 min after the treatments (Henriques et al., 1990). After 4 h, the animals were euthanized for collection of pleural exudates in 1 mL of PBS-EDTA. Subsequently, samples were centrifuged at 2500 rpm for 10 min, the supernatants were discarded, and the pellets were resuspended in 0.5 mL PBS-EDTA. Aliquots of 50 µL of samples were taken for counting total leukocytes in a Neubauer chamber.

### 2.9. Zymosan-Induced Arthritis Model

Female Swiss mice were distributed into 6 groups of 5 animals each. The control and naïve groups were treated with saline (0.9%), the treatment group was treated with EEPV 700 mg/kg orally, and the reference group received dexamethasone at 1 mg/kg i.p. After 60 min of treatment, all animals received 20 µL injections of zymosan (500 µg/articular cavity) behind the suprapatellar tendon of the left knee space [10,11]. After 4 and 6 h of induction, the animals had the edema volume measured using a digital micrometer; in these times, the mechanical hyperalgesia was measured using a digital analgesimeter (Von Frey). After 6 h of arthritis induction, the animals were euthanatized, and knee joints were exposed by surgical incision and washed twice with 5 µL of PBS/EDTA. The exudate was diluted to a final volume of 50 µL with PBS/EDTA to determine the total leukocyte counts. The total number of leukocytes was determined in a Neubauer chamber using a light microscope. The results were expressed as the number of leukocytes per articular cavity [12].

### 2.10. In Situ Intravital Microscopy Analysis for Rolling and Adhesion Events of Leukocytes in the Mesenteric Microcirculation

The mice received orally 300 mg/kg of EEPV, vehicle (10% Tween solution) or indomethacin (5 mg/kg), 30 min prior the carrageenan injection (*n* = 5–7 animals/group). One additional mouse group (negative control) was treated only with saline solution in the peritoneal cavity. The animals were anesthetized with ketamine/xylazine solution (1:1), the carrageenan injection was performed, and the control and EEPV groups received an injection of 500 µg/cavity by the intraperitoneal route (i.p.). Subsequently, the intravitreal microscopy analysis was performed two hours after carrageenan intraperitoneal or saline (naïve) injection. A lateral incision was performed in the abdominal wall of mice to exposure of the mesentery to the observation of in situ microcirculation. The mice were maintained on a heated plate (37 °C) adapted to observation of optical microscopy with a video camera/monitor to project and record the images. The preparation was kept moist and warm with Ringer Locke’s solution (pH 7.2–7.4), which contained 1% gelatin. The vessels were considered the postcapillary venules with 10–18 µm diameter. During 10 min, the number of rolling and adherent leukocytes of each animal was recorded. Leukocyte adherence was determined when leukocyte remained static in the endothelium for more than 30 s.

### 2.11. Paw Edema, Mechanical Hyperalgesia, Cold Response, Myeloperoxidase (MPO), and Enzyme N-acetilglucosaminidase (NAG) Analysis in CFA-Induced Paw Inflammation for 24 h

Three groups of five female mice received two oral administrations (p.o.) (the first oral administration at time 0 and the second exposure after 24 h) of saline solution (0.9%, control group), EEPV (300 mg/kg, p.o.), and the positive control received dexamethasone 1 mg/kg (s.c). After the first administration, the animals received 20 μL of oil suspension containing killed *M. tuberculosis* (Freund’s complete adjuvant—CFA) by intraplantar injection in the right paw. After 3, 4, and 24 h of CFA administration in the paw, cold sensitivity was measured after acetone stimulation, mechanical hyperalgesia was analyzed by the von Frey method, while paw edema was measured with a plethysmometer (Insight^®^). Paw edema, MPO, and NAG activity were analyzed 24 h after CFA injection. The migration of neutrophils and macrophages to the skin tissue of the paw in female mice was indirectly quantified through the activity of MPO and NAG, respectively. For this purpose, the skin of the paw of female mice were removed 24 h after the CFA injection. All the descriptions of these methodologies were performed according to Formagio et al. (2022) [13].

### 2.12. Rota Rod Test

Two groups of five female mice received one single administration of saline solution (0.9%, p.o., control group) and EEPV (300 mg/kg, p.o.). After 60 minutes, mice were placed on a horizontal rod that rotates about its long axis; the animal must walk forwards to remain upright and not fall off [14].

### 2.13. Statistical Analysis

The data are presented as the mean ± standard error (SEM). The determination of significant differences among groups was made via one-way analysis of variance (ANOVA) and the Tukey test were chosen as a post hoc (GraphPad Prism Software version 8.0, La Jolla, CA, USA). The percentage of inhibition was calculated from the control group. Differences were considered to be significant when *p* < 0.05.

## 3. Results

### 3.1. Phytochemical Analysis

The analyzes of the chemical content of EEPV indicated a content of phenolic compounds of 58.45 mg/g and of flavonoids of 12.03 mg/g.

### 3.2. Analysis of EEPV by HPLC-DAD

**Figure 1:**
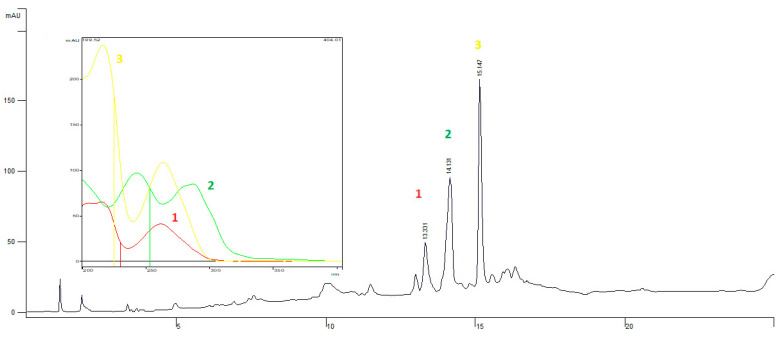
Chromatogram (HPLC-DAD) of the EEPV. Peak 2 (green) is a flavonol, and peaks 1 (red) and 3 (yellow) are substances of the amide class.

Figure 1 shows the chromatogram and UV spectra of the ethanol extract, and the UV spectra indicate that the substances present in the extract are of different classes. Substance 2, with retention time 14.131, is a flavonol [15], and substances 1 and 3, with retention times 13.331 and 15.147, are from the amide class [16].

### 3.3. EEPV Inhibited Nociceptive Response in Phase 1 and 2 and Cold Allodynia in Formalin Model

**Figure 2:**
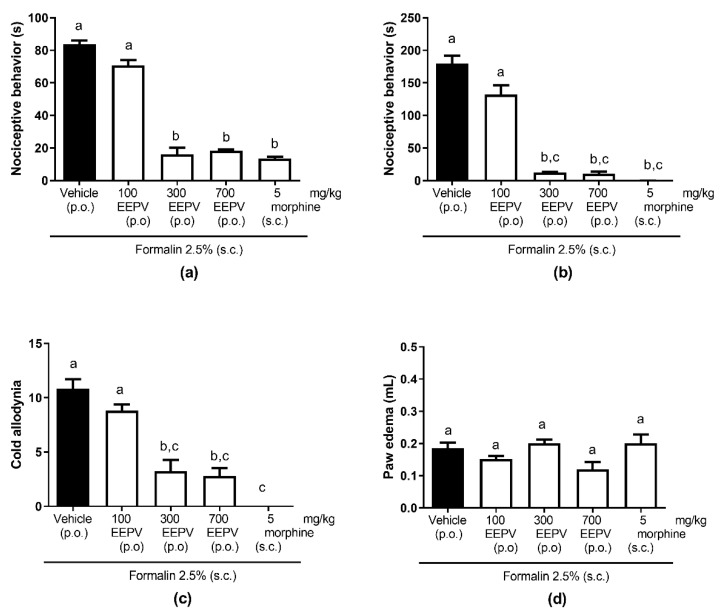
Effect of treatment with EEPV in a model of formalin-induced nociception. The animals were treated with 100, 300, or 700 mg/kg; vehicle group orally received saline solution (0.9%); the morphine group received subcutaneous doses of 5 mg/kg (*n* = 5–7 animals/group). Antinociceptive activity observed through phases I (**a**) and II (**b**). Sensitivity to cold with acetone after 30 min of intraplantar formalin injection (**c**). Edema evaluated 1 h after intraplantar injection of formalin 2.5% (**d**). Each bar represents the mean ± SEM. The letters “a”, “b”, and “c” indicate significant differences among groups according to Tukey´s multiple comparisons test.

In phase 1, the oral EEPV administration of 300 and 700 mg/kg induced significant nociceptive inhibition (81% and 78%, respectively); however, the dose of 100 mg/kg did not differ from the control group results (Figure 2a). In phase 2, the oral EEPV administration of 300 and 700 mg/kg induced significant nociceptive inhibition (93% and 68%, respectively); however, the dose of 100 mg/kg did not differ from control group results (Figure 2b). None of the doses of the EEPVs tested showed antiedematogenic properties (Figure 2c). In the cold response test, the inhibition results were 70% and 74% for 300 and 700 mg/kg of the EEPV, respectively. The morphine group inhibited the nociceptive response in phase 1, 2, and also the cold response (Figure 2d). The comparison among the groups showed that the EEPV had similar patterns of response in relation to phase 1, phase 2, and the cold response. The dose of 300 and 700 mg/kg of the EEPV did not differ between themselves; however, they were different from the control and the 100 mg/kg of EEPV groups (Figure 2a–c).

### 3.4. EEPV Inhibited Abdominal Writhing Induced by Acetic Acid

**Figure 3:**
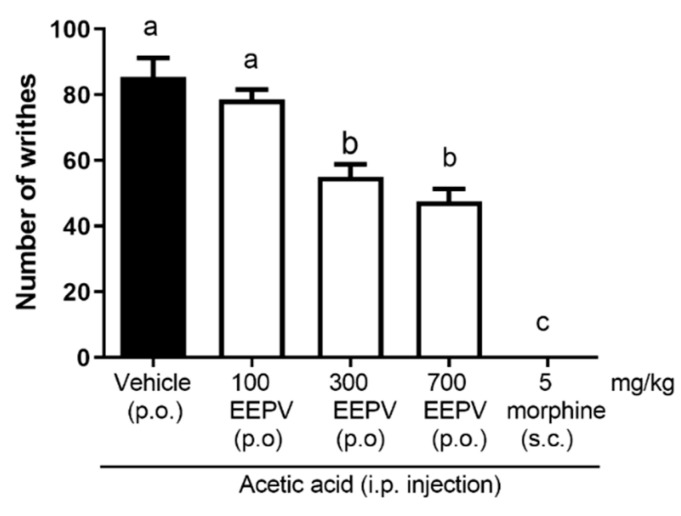
Effect of EEPV treatment on acetic acid-induced abdominal writhing. The animals were treated with 100, 300, or 700 mg/kg; vehicle group received orally saline solution (0.9%); the morphine group received subcutaneous doses of 5 mg/kg (*n* = 5–7 animals/group). After injection of acetic acid 0.8% i.p., the count of the number of writhings. Bars represent mean ± SEM. The letters “a” and “b” indicate significant differences among groups according to Tukey´s multiple comparisons test.

In the analysis of the antinociceptive effect of EEPV on acetic acid-induced abdominal writhing, a significant effect was observed at both doses (300 and 700 mg/kg), 36% and 44%, respectively. The dose of 100 mg/kg of EEPV did not differ from control results. The morphine group showed total inhibition of the effect (Figure 3). In abdominal writhing induced by acetic acid, the comparison among groups showed that the doses of 300 and 700 mg/kg of EEPV did not differ between themselves; however, they were different from control and the 100 mg/kg of the EEPV groups (Figure 3).

### 3.5. EEPV Reduced Leukocyte Migration in Carrageenan-Induced Pleurisy Model

**Figure 4:**
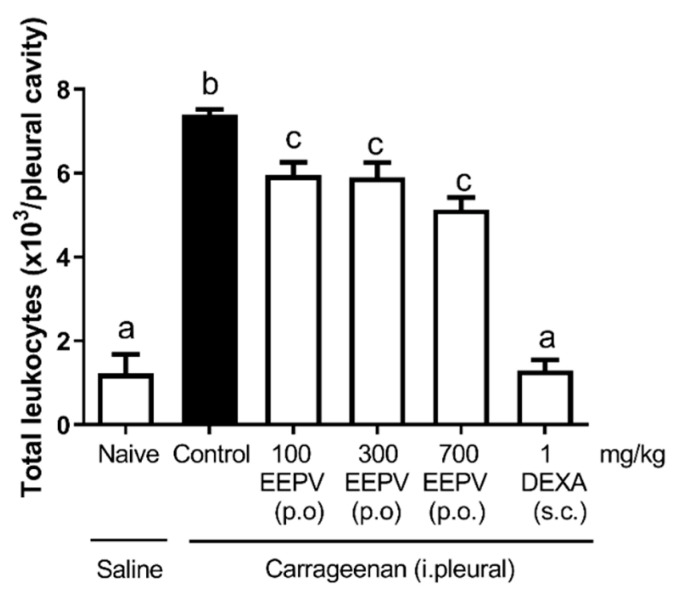
Evaluation of leukocyte recruitment in a carrageenan-induced pleurisy model. Treatment with EEPV at 100, 300, and 700 mg/kg, vehicle, and dexamethasone at dose of 1 mg/kg (*n* = 5–7 animals/group). Each bar represents the mean ± SEM. The letters “a”, “b”, and “c” indicate significant differences among groups according to Tukey´s multiple comparisons test.

Four hours after intrapleural carrageenan injection, the EEPV group at doses of 100, 300, and 700 mg/kg decreased the leukocyte migration (Figure 4). All three treated groups differed statistically from the control and from naïve groups. The leukocyte recruitment inhibition was: 20.27% for the EEPV group at a dose of 100 mg/kg, 19.45% at a dose of 300 mg/kg, 26% at a dose of 700 mg/kg, and 81% for the DEXA group (Figure 4). All EEPV-treated groups did not differ statistically among themselves; however, all treated groups differed from the control.

### 3.6. EEPV Reduced Knee Edema, Mechanical Hyperalgesia, Leukocyte Migration in Zymosan-Induced Articular Inflammation

**Figure 5:**
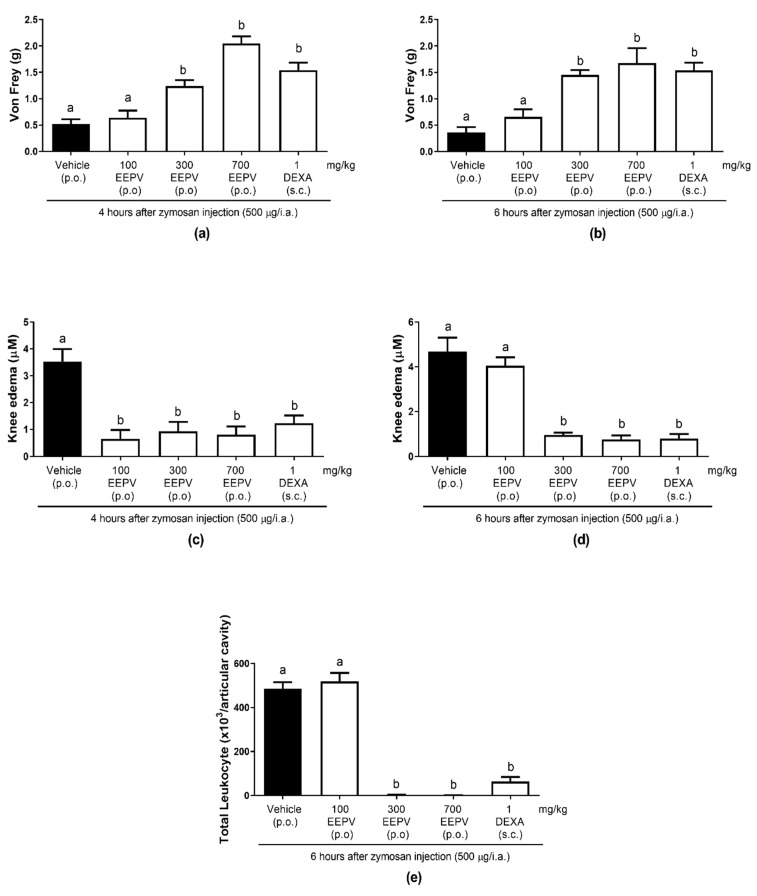
Effect of oral administration of EEPV on the zymosan-induced knee edema, mechanical hyperalgesia, leukocyte migration in synovial exudate in mice. The animals received EEPV (100, 300, and 700 mg/kg, p.o.), vehicle (control), or dexamethasone (DEXA, 1 mg/kg, s.c.) (*n* = 5–7 animals/group), and 1 h later, an intraplantar injection of zymosan was administered. Graphs (**a**,**b**) represent the evaluation of the mechanical hyperalgesia at 4 and 6 h, respectively, after zymosan injection; graphs (**c**,**d**) represent the evaluation of the knee edema at 4 and 6 h, respectively, after zymosan injection; graph (**e**) represents the evaluation of the total leukocytes counts 6 h after stimulus. The letters “a” and “b” indicate significant differences among groups according to Tukey´s multiple comparisons test.

After 4 h of zymosan administration in the knee, oral exposure of female mice to a dose of 300 mg/kg of EEPV prevented mechanical hyperalgesia in 82.66%, while the dose of 700 mg/kg of the EEPV and the treatment with dexamethasone blocked the mechanical response (Figure 5a). After 6 h of zymosan injection in the knee, the doses of 300 and 700 mg/kg of the EEPV and the dexamethasone group blocked the development of mechanical hyperalgesia (Figure 5b). After 4 h of zymosan administration in the knee, all doses of the EEPV tested and dexamethasone groups interfered statistically in knee edema when compared with the control group (Figure 5c). The inhibition of knee edema induced by EEPV was 81.58% for 100 mg/kg, 73.65% for 300 mg/kg, 77.33% for 700 mg/kg, while for dexamethasone, it was 62.15%. After 6 h of zymosan administration in the knee, the oral administration of 300 mg/kg of EEPV prevented the knee edema in 79.71%, while the dose of 700 mg/kg prevented of 83.81% and the dexamethasone prevented 83% (Figure 5d). In relation to total leukocyte migration to the knee, the doses of 300 and 700 of the EEPV blocked the migration of these cells to synovial liquid, while the dexamethasone inhibited 86.8% (Figure 5e). The oral exposure to the doses of 300 and 700 mg/kg of EEPV in did not differ among themselves; however, they differed from the control and from the dose of 100 mg/kg of the EEPV (Figure 5a,b,d,e). The mechanical hyperalgesia, cold response, and knee edema (6 h) evaluation showed that the groups treated with doses of 300 and 700 mg/kg of EEPV did not differ among themselves; however, they differed from the control and from the dose of 100 mg/kg of the EEPV (Figure 5a,b,d,e).

### 3.7. EEPV Reduces Rolling Leukocytes and Leukocyte Adhesion on Mesenteric In Situ Microcirculation

**Figure 6:**
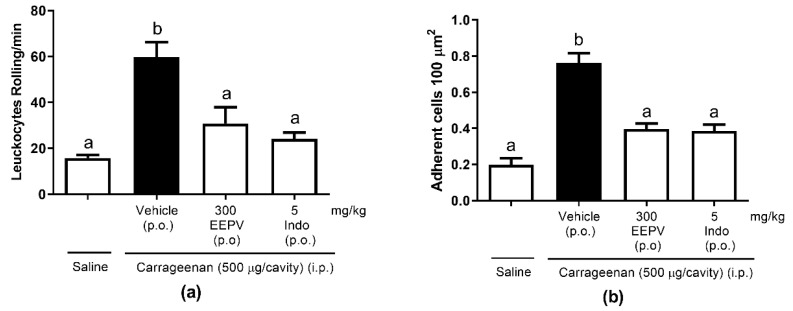
Effect of EEPV on leukocyte rolling (**a**) and adhesion (**b**) induced by carrageenan. Mice were orally pretreated with EEPV (300 mg/kg), indomethacin (indo, 5 mg/kg), or vehicle (*n* = 5–7 animals/group). After 60 min, saline or carrageenan was injected i.p. Leukocyte rolling and adhesion were evaluated by intravital microscopy in the mesentery 2 h later. Results are expressed as mean ± S.E.M. and are representative of three independent experiments. The letter “a” indicates significant differences between the control and saline group, and letter “b” indicates significant differences among the control group, the EEPV, and indomethacin groups according to the one-way ANOVA, followed by the Tukey test.

Carrageenan injection (i.p.) significantly increased rolling (Figure 6a) and adhesion (Figure 6b) of leukocytes in the endothelium 2 h after inflammatory stimulation when compared to the control group. Oral pretreatment with the EEPV (300 mg/kg) significantly diminished leukocyte rolling by 48.84% and adhesion by 48.2%. compared to the control group. Indomethacin, used as reference drug, reduced leukocyte rolling by 59.67% and adhesion by 49.51% compared to control group.

### 3.8. EEPV Reduces Mechanical Hyperalgesia, Cold Sensitivity, Edema Formation, Myeloperoxidase (MPO), and Enzyme N-acetilglucosaminidase (NAG) Activity in CFA-Induced Inflammation

**Figure 7:**
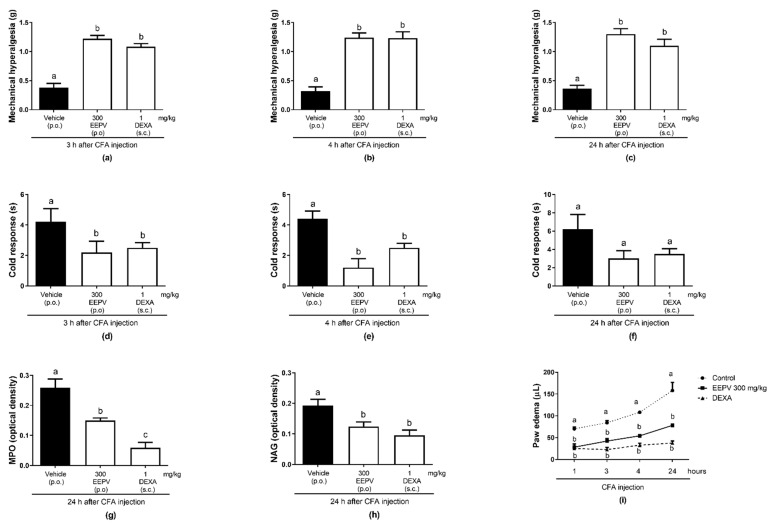
Effect of oral administration of EEPV on mechanical hyperalgesia and response to cold after 3 (**a**,**d**), 4 (**b**,**e**), 24 h (**c**,**f**), and MPO (**g**), NAG (**h**), and edema (**i**) after CFA injection in mice. Mice were treated two times (time 0 and 24 h from the first treatment) with EEPV (300 mg/Kg p.o.), dexamethasone (DEXA—1 mg/kg, s.c.), or vehicle (*n* = 5–7 animals/group). The bars express the mean ± SEM. Differences between groups were analyzed by analysis of variance (one-way ANOVA) followed by the Tukey test. The letters “a”, “b” and “c” indicate significant differences between groups.

After 3, 4, and 24 h of the CFA administration in the paw, oral exposure of female mice to a dose of 300 mg/kg of the EEPV and the treatment with dexamethasone blocked the mechanical hyperalgesia response (Figure 7a–c). After 3 h of the CFA administration in the paw, the oral administration of 300 mg/kg of the EEPV prevented the cold response in 47.61%, while dexamethasone prevented 40.47% (Figure 7d). After 4 h of the CFA administration in the paw, the oral administration of 300 mg/kg of the EEPV prevented the cold response in 72.72%, while dexamethasone prevented 56.81% (Figure 7e). After 24 h of the CFA administration in the paw, the oral administration of 300 mg/kg of the EEPV prevented the cold response in 51.6% while dexamethasone prevented 43.5% (Figure 7f). After 24 h of the CFA administration in the paw, the oral administration of 300 mg/kg of the EEPV prevented MPO activity response in 42.22%, while dexamethasone prevented 77.18% (Figure 7g). After 24 h of the CFA administration in the paw, the oral administration of 300 mg/kg of the EEPV prevented NAG activity response in 35.98%, while dexamethasone prevented 50.98% (Figure 7h). In edema, the EEPV was effective in the inhibition at 3, 4, and 24 h induced by the CFA (Figure 7i).

## 4. Discussion

To the best of our knowledge, this is the first report of the antiarthritic and antinociceptive effects of ethanolic extract of *Piper vicosanum* leaves (EEPV) on in vivo models of articular inflammation and nociceptive models (formalin-induced nociception test and acetic acid-induced abdominal writhing test). Few studies have demonstrated the pharmacological potential of *P. vicosanum*, and our research group has focused on demonstrating the safety of this plant in the most recent articles published on this subject [4,17]. To our knowledge, only the anxiolytic [7] and anti-inflammatory [6] activity of *P. vicosanum* oil has been investigated. The results of joint inflammation and CFA-induced inflammation models indicate an antiarthritic potential of the EEPV, while the in situ microcirculation results demonstrate that the EEPV-induced inhibition of migration plays an important role in this mechanism. The analgesic effect of the EEPV was shown in the models of the formalin-induced nociception, acetic acid-induced nociception, and also in mechanical and thermal hyperalgesia in the CFA and zymosan models.

The chemical analysis of the EEPV described that this extract contains alkaloid, phenolic, tannin, steroid, and triterpenoid compounds [7]. Morphine analogues and alkaloids obtained from medicinal plants represent important sources of new drug analgesic candidates [18]. Phenolics and its derivatives also possess relevant biological properties [19], while tannins may be employed as a therapeutic agent [20]. Several steroids administered in patients are effective in decreasing postoperative pain and in the reduction of inflammatory markers [21]. The presence of alkaloid, phenol, tannin, steroid, and triterpenoid compounds in the EEPV could contribute to the biological activity found in the *P. vicosanum* specimen.

In relation to the analgesic effects of the EEPV, no studies were found concerning the analgesic properties of *P. vicosanum* in the published literature. The formalin model could show the analgesic potential of products in phase 1 and 2 in relation to spontaneous pain. In the present work, the doses of 300 and 700 mg/kg of the EEPV showed antinociceptive properties in both phases, showing that the EEPV action is involved in the antagonization of inflammatory and neurogenic pain. Frequently, the majority of nonsteroidal anti-inflammatory drugs (NSAIDs) only inhibit phase 2 without interfering with phase 1. Morphine inhibits both phases, and the analgesic effects of the EEPV could be similar to these opioid effects. The EEPV also inhibited the abdominal contortion induced by acetic acid (Figure 3). The abdominal contortion model is a different type of experimental assay to verify the antinociceptive effects of medicinal plants [22,23,24,25]. The rotarod test was performed in mice to test if EEPV induced alterations in motor coordination [14]. When the animals are placed on a Rota rod apparatus, they did not fall, showing that the EEPV did not alter motor conditions (results not shown).

Animal models in pain research, such as the complete Freund’s adjuvant (CFA) inflammatory pain in mice, included both evoked and nonevoked behavioral measurements, and it could reflect the human pain experience [26]. The CFA-induced paw inflammation model was chosen since it causes an inflammatory pain characterized by mechanical and thermal stimulation. Our results showed that EEPV is able to inhibit both mechanical and cold nociceptive responses (after 3, 4, and 24 h from CFA injection) stimulated by von Frey apparatus and by acetone, respectively (Figure 7a–f). In addition to this aspect, the CFA-induced chronic inflammation model simulates an arthritis-like inflammation.

The MPO activity measured the neutrophil migration while the NAG activity analyzed the monocyte migration, both indirectly. The EEPV inhibited these enzyme activities, showing a reduction of the migration of neutrophils and monocytes. In the present study, it was possible to reveal that acute exposure to the EEPV inhibited all parameters (mechanical hyperalgesia, cold allodynia, edema, MPO, and NAG activity) (Figure 7a–g) elicited by the CFA injection into the paw, corroborating the data of zymosan-induced inflammatory articular pain. These results confirm the analgesic, anti-inflammatory, and antiarthritic potential of *P. vicosanum*, and also showed that the EEPV reduces both neutrophil and monocyte migration to inflammatory sites in the CFA inflammatory model in mice.

Two hours after the carrageenan injection, the phenomenon of leukocyte rolling/adhesion to the mesentery was observed by intravital microscopy. The EEPV reduced the leukocyte–endothelium interaction (Figure 6) and also the leukocyte transmigration (Figure 4). In our results, the efficacy of the oral EEPV was similar to the oral indomethacin treatment. Although the doses were different, the inhibition values did not differ statistically when the groups were compared. The results showed that the EEPV is effective against leukocyte/adhesion in inflamed mesenteric microcirculation, showing that this oil may affect adhesion molecules.

Branquinho et al. (2017) [27] showed the antiedematogenic effects of essential oil from *P. vicosanum* leaves (100 and 300 mg/kg) on a carrageenan-induced paw edema model, and also demonstrated the inhibition of leukocyte migration in a pleurisy model. In the present study, the effects of the EEPV on leucocyte inhibition was shown with doses of 100, 300, and 700 mg/kg (Figure 4). The murine model of zymosan-induced arthritis is a pharmacological tool used to study several inflammatory mechanisms and is useful as an experimental model to assess the anti-inflammatory effects of different products [10]. In the beginning of the inflammatory process induced by zymosan, this inflammatory agent stimulated an important oedema formation, accompanied by a massive neutrophil infiltration in the synovial tissue and the fluids of the inflamed joints [28]. The present study showed that intraarticular zymosan injection in mice resulted in an expressive increase in leukocyte migration, in knee joint thickness, and in mechanical hyperalgesia (Figure 5).

The EEPV, mainly in doses of 300 and 700 mg/kg, showed a high efficacy against the mechanical hyperalgesia development (Figure 5a,b), knee edema formation (Figure 5c,d), and leukocyte migration (Figure 5e) induced by zymosan. The EEPV had similar or higher efficacy when compared to dexamethasone in the inhibition of these parameters (Figure 5a–e). Experimental studies have demonstrated the inhibition of leukocyte recruitment to the knee joint constitutes a useful therapeutic strategy to treat rheumatoid arthritis [29]. The antiarthritic efficacy of the treatment of female mice with a dose of 100 mg/kg was different from the results of mice treated with doses of 300 and 700 mg/kg. These results suggested that the EEPV is an antiarthritic agent, and this action indicated that this extract acts on specific targets.

## 5. Conclusions

For the first time, the analgesic, anti-inflammatory and antiarthritic potential effects of the EEPV was revealed in inflammation, arthritis, and nociception models. Our data support the antiarthritic activity in terms of the inhibition of leukocyte migration, characterized by reduction of leukocyte infiltration, adhesion, and rolling. Antinociceptive activity was also observed in formalin and in acetic acid-induced abdominal writhing models. Taken together, the data suggest that the EEPV has therapeutic potential in arthritis and pain diseases.

## Data Availability

All data are available in this publication.
